# American Institute of Homœopathy

**Published:** 1896-12

**Authors:** Joseph T. Cook, Chas. L. Mosher

**Affiliations:** Secretary Local Committee, 636 Delaware Ave., Buffalo, N. Y.; Secretary Local Committee, 636 Delaware Ave., Buffalo, N. Y.


### AMERICAN INSTITUTE OF HOMŒOPATHY.

BUFFALO, N. Y., November 14th, 1896.

EDITOR OF THE HOMŒOPATHIC PHYSICIAN:

The decision of The American Institute of Homœopathy to hold its next meeting in Buffalo, N. Y., was received with much pleasure by the profession in this city.

Active steps were at once taken by Dr. A. R. Wright, who had been appointed Chairman of the Local Committee of Arrangements, toward choosing his assistants, and forming the eleven sub-committees to have charge of the several branches of the work to be accomplished.

The sub-committees, composed of about six persons in each, were completed in August last, and have already made material advancement in their respective departments.

Buffalo has gained much celebrity of late as a convention city: no less than twenty National Associations having met there this season. Many more are looked for next year, including the Encampment of the G. A. R., which will bring to the city no less than 300,000 persons, including delegates and their friends.

For the American Institute, which will meet in Buffalo in June of next year, the Local Committee have already engaged the Iroquois Hotel as headquarters, and also have arranged, at the same hotel, for several committee rooms.

It has been suggested that the alumni associations of the various medical colleges may desire to engage headquarters for their societies during this meeting, and it would be well for such to report to the Local Committee in good season, in order to obtain desirable locations.

A special feature of the work of the Local Committee will be that done by the Sub-committee on New Members; working in connection with the regular Committee of the Institute. A particular effort will be made to increase the membership. It is proposed to send an urgent invitation to every homœopathic physician in the United States, who is not now a member, asking him to join this year.

Further details of the efforts of the Local Committee will be announced as the work progresses.

Fraternally yours,

JOSEPH T. COOK,
Per CHAS. L. MOSHER,
*Secretary Local Committee,*
636 Delaware Ave., Buffalo, N. Y.

By order of Dr. A. R. Wright, Chairman Local Committee
414 Elmwood Avenue, Buffalo, N. Y.

